# Comparative Effectiveness of Multiple Psychological Interventions for Psychological Crisis in People Affected by Coronavirus Disease 2019: A Bayesian Network Meta-Analysis

**DOI:** 10.3389/fpsyg.2021.577187

**Published:** 2021-02-22

**Authors:** Yang Yang, Shaodan Sun, Shaowen Hu, Chunzhi Tang, Yimin Zhang, Haibo Lin

**Affiliations:** ^1^Affiliated Jiangmen Traditional Chinese Medicine Hospital of Jinan University, Jinan University, Guangzhou, China; ^2^Integrated Chinese and Western Medicine Postdoctoral Research Station, Jinan University, Guangzhou, China; ^3^Department of Pharmacology of Traditional Chinese Medicine, The Second Affiliated Hospital of Guangzhou University of Chinese Medicine, Guangzhou, China; ^4^The Postdoctoral Research Station, Guangzhou University of Chinese Medicine, Guangzhou, China; ^5^Shenzhen Bao'an Traditional Chinese Medicine Hospital Group, Guangzhou University of Chinese Medicine, Shenzhen, China; ^6^Clinical Medical College of Acupuncture and Rehabilitation, Guangzhou University of Chinese Medicine, Guangzhou, China; ^7^School of Traditional Chinese Medicine, Jinan University, Guangzhou, China

**Keywords:** psychological intervention, COVID-19, affected people, psychological crisis, network meta-analysis

## Abstract

**Objective:** The objective of our current research is to compare the different psychological interventions and distinguish the most effective way to treat psychological crisis according to different clinical manifestations in people affected by coronavirus disease 2019 (COVID-19). No previous systematic review has provided a comprehensive overview by performing a Bayesian network meta-analysis of this current topic.

**Method:** A systematic review and a Bayesian network meta-analysis were conducted on randomized controlled trials (RCTs), non-RCTs, case–control studies, self-controlled case series (SCCS), cohort studies, and cross-sectional studies of all the available interventions for psychological crisis in people affected by COVID-19. We searched the electronic databases EMBASE, PubMed, Web of Science, PsycINFO, and Cochrane Library, as well as the Chinese databases such as Sinomed, Chinese Biomedicine Literature (CBM), Chinese Scientific Journal Database (VIP), WanFang Database, and China National Knowledge Infrastructure (CNKI), from 2019 to April 30, 2020. The main outcomes were self-rating anxiety scale (SAS), self-rating depression scale (SDS), patient health questionnaire (PHQ-9), and symptom checklist (SCL-90). The study is registered with Inplasy, number 202050076.

**Result:** Sixteen self-controlled case series (SCCS) comprising 1,147 participants compared five different psychological interventions with four different measurement scales were included in this study. For effectiveness, all the psychological therapies were significantly more effective than before intervention. Our results showed that supportive therapy (ST), which is adjusted to the COVID-19-related mental crisis, is the best treatment compared with behavioral therapy (BT), nursing-based psychological therapy (NBPT), traditional Chinese medicine therapy (TCMT), and COVID-19-related standard training (CRST) at reducing the anxiety-related symptoms assessed by SAS. When measured by SDS, BT was better than ST and NBPT treatment for reducing the depression symptoms. And ST was better than BT and ST+BT as assessed by PHQ-9. In the end, the last network meta-analysis indicated that NBPT was more effective than ST by the measurement of SCL-90.

**Conclusion:** Our research suggested the potential effectiveness of psychological interventions for decreasing psychological crisis in people affected by COVID-19 and try to introduce the best effective treatment options for clinical practice according to the clinical manifestations of psychological problems, but further confirmation from high-quality RCTs is needed.

## Introduction

The acute respiratory infectious disease caused by the outbreak of coronavirus disease 2019 (COVID-19) spread quickly to all parts of the world. The World Health Organization (WHO) points out that the COVID-19 is an international public health emergency with the highest mortality rate among the new-onset infectious diseases (Sohrabi et al., 2020). The outbreak occurred during the Chinese New Year. The high mobility of the population is very conducive to the spread of the virus, resulting in a rapid increase in the number of affected people, which poses a great threat to human health, resulting in extremely tight medical resources and immense psychological pressure on both medical staff and patients (Blake et al., 2020; Talevi et al., 2020).

Due to the impact of the epidemic, many changes have taken place in people's daily lives. Life seems to be filled with information related to the epidemic. There has also been a panic reaction of irrationally hoarding food, snapping up masks, and disinfecting supplies. Many studies have found that COVID-19 patients and medical staff are more prone to mental disorders than the general population, such as feeling uneasy, worried, fear, confused, and helpless; insomnia; depression; and other psychological crises (Petzold et al., 2020; Wu and Zhang, 2020).

Psychological intervention is aimed to reduce the risk of acute psychological crisis and stabilize or reduce the direct and serious consequences of psychological crisis on the individual, thereby promoting the individuals to recover from the crisis. After the outbreak, whether or not to take correct measures in a timely manner is an important factor in rehabilitation. We believe that actively carrying out mental health work on such people can reduce the potential and long-term impact on the mind.

At present, Internet information is convenient and well-developed; various stress manuals, methods of psychological intervention (including professional intervention and self-intervention), video, audio, and WeChat articles are overwhelming. Information overload makes many effective psychological intervention methods submerged in a large amount of information. And which psychological intervention is better is still controversial. Therefore, finding effective psychological intervention is particularly important. Based on this, the Bayesian method is used here to analyze the therapeutic effects of different psychological interventions and to explore the best psychological intervention methods under the COVID-19 epidemic.

We compared five different psychological interventions in this research. First, supportive therapy (ST) is a commonly used and well-developed psychological intervention with a long history, and the studies we included in this article were adjusted according to the particularity of the COVID-19 epidemic. Second, BT is another commonly used non-pharmacological application defined as behavior-change intervention, including exercise or changes in daily activity to help deal with the psychological problems. Third, traditional Chinese medicine therapy (TCMT) is a psychological adjustment method based on traditional Chinese medicine theory and modern psychology, such as acupoint-plucking emotional freedom method (Tian, 2014). Next, COVID-19-related standard training (CRST) refers to the training of medical expertise in the guidelines related to the COVID-19, as well as learning to deal with psychological crisis caused by COVID-19. The last one, nursing-based psychological therapy (NBPT), is a unified nursing process with characteristics formulated according to the COVID-19 treatment plan, including breath training and psychological evaluation and guidance. We hope to provide scientific and effective psychological intervention methods for maintaining the mental health of people affected by COVID-19.

## Methods

We conducted this study according to the Cochrane Handbook for the Systematic Review of Interventions (see details at http://training.cochrane.org/handbook), and reporting was in line with the Preferred Reporting Items for Systematic Reviews and Meta-Analyses (PRISMA) extension statement for network meta-analyses (Liberati et al., 2009). Included studies were classified according to the types of psychological interventions.

### Search Strategy

We searched the electronic databases EMBASE, PubMed, Web of Science, PsycINFO, and Cochrane Library, as well as Chinese databases such as Sinomed, Chinese Biomedicine Literature (CBM), Chinese Scientific Journal Database (VIP), WanFang Database, and China National Knowledge Infrastructure (CNKI), from 2019 to April 30, 2020. Searches were not restricted by language. We aimed to compare all psychological interventions used for psychological crisis in people affected by COVID-19 (see Supplementary Figure 1 for full search terms).

### Study Selection

#### Participants

Psychological crisis was assessed in people affected by COVID-19, which includes confirmed patients, patients with suspected infection, quarantined relatives, and other patients who have a high risk of infection due to other diseases that have to be treated in the hospital, as well as caregivers and health-care professionals, such as doctors, nurses, and health-related administrators.

#### Interventions

All types of psychological interventions were included as long as the explicit aim was to prevent anxiety, depression, and fear of any other type of psychological crisis.

#### Comparisons

Any type of psychological treatments was compared with each other or with other control groups (placebo, blank, and usual care) who were eligible.

#### Outcomes

At least one outcome reported psychological symptoms. The primary outcomes were self-rating anxiety scale (SAS), self-rating depression scale (SDS), patient health questionnaire (PHQ-9), and the symptom checklist (SCL-90), which were also analyzed by a network meta-analysis.

#### Study Design

Randomized controlled trials (RCTs), non-RCTs, case–control studies, self-controlled case series (SCCS), cohort studies, and cross-sectional studies were all included.

#### Exclusion Criteria

(1) The same patients were enrolled in different articles; (2) duplicate reports, conferences, observational studies (prospective and retrospective), review articles, nonhuman studies, studies with incorrect comparator, and case reports were strictly excluded.

### Data Extraction and Quality Assessment

Two investigators (YY and SWH) independently selected the studies. The extractions of the relevant information from the included trials were extracted with a predetermined data extraction sheet (Table 1). The risk of bias assessments was performed at the outcome measure level during data collection. And different types of tool were used according to the different study designs. Any disagreements were resolved through discussion. When they could not reach a consensus, the final decision regarding each question was made by other investigators (HBL) within the review team.

**Table 1:** Baseline of included studies.

**Year**	**First author**	**Location**	**Participants**	**Mean age in years**	**Sex**	**Intervention**	**Main outcomes**	**Final sample size**	**Duration**
2020	Man-Ping Zeng (Zeng et al., 2020)	Hunan, China	Nurse	29 ± 3	M/F 5/37	TCMT	SAS	37	10 days
2020	Xiao-Ping Huang (Huang and Ke, 2020)	Guangdong, China	Hospital disinfection supply center staff	38.28 ± 11.9	M/F 18/32	CRST	SAS	50	1 week
2020	Wei Mi (Mi and Yu, 2020)	Anhui, China	Confirmed patients	39.05 ± 13.22	M/F 10/10	NBPT	SAS	20	2 weeks
2020	Xue-Ying Li (Li and Tang, 2020)	Hubei, China	Confirmed patients	No mention	M/F 23/21	NBPT	SAS, SDS	48	5 days
2020	Hong Chen (Chen et al., 2020)	Hubei, China	Confirmed patients	51.55 ± 18.36	M/F 39/36	BT	SF-36, SAS, SDS	75	From admission to discharge
2020	Xia Xu (Xu, 2020)	Hubei, China	Confirmed patients	No mention	No mention	BT	PHQ-9	208	1 week
2020	Ying Ren (Ren et al., 2020)	Henan, China	Doctor	No mention	M/F 15/39	ST	SCL-90	54	1 week
2020	Xia Li (Li et al., 2020)	Hubei, China	Nurse	30.32 ± 5.39	M/F 13/108	ST	GAD-7, PHQ-9, PSQI	121	1 week
2020	Chun-Yan Kuang (Kuang et al., 2020)	Guangdong, China	Confirmed patients	35.2 ± 9.66	M/F 32/36	ST	SAS, SDS	68	2 weeks
2020	Li-Min Xing (Xing et al., 2020)	Hubei, China	Nurse	31.37 ± 7.26	M/F 2/38	NBPT	SCL-90	40	2 weeks
2020	Yan-Li Yang (Yang et al., 2020)	Guangdong, China	Other inpatient	51.2 ± 4.3	M/F 32/18	NBPT	SAS	50	1 week
2020	Cui Tian (Tian et al., 2020)	Beijing, China	Nurse	26.75 ± 3.67	M/F 3/57	ST+BT	GAD-7, PHQ-9, PSQI	60	1 week
2020	Yan-Qiao Bao (Bao et al., 2020)	Hubei, China	Nurse	No mention	M/F 11/34	NBPT	SCL-90	45	2 weeks
2020	Yan-Wen Dong (Dong, 2020)	Hubei, China	Health-related administrators	25–55	M/F 19/37	ST	SAS, SDS	56	No mention
2020	Xuan Zhou (Zhou et al., 2020)	Zhejiang, China	Nurse	33.27 ± 7.43	M/F 10/195	ST	SAS, SDS	205	1 week
2020	Yang Zhang (Zhang et al., 2020)	Zhejiang, China	Confirmed patients	44.9 ± 19.2	M/F 5/5	ST	SAS, SDS	10	1 time

*TCMT, traditional Chinese medicine therapy; SAS, self-rating anxiety scale; CRST, coronavirus disease 2019-related standard training; NBPT, nursing-based psychological therapy; SDS, self-rating depression scale; BT, behavioral therapy; SF-36, 36-Item Short Form Health Survey; PHQ-9, patient health questionnaire; ST, supportive therapy; SCL-90, symptom checklist; GAD-7, General Anxiety Disorder-7; PSQI, Pittsburgh Sleep Quality Index*.

### Statistical Analysis

First, we summarized and analyzed the baseline data and outcomes of involved studies' characteristics. Accordingly, mean differences (MDs) for continuous outcomes with 95% credible intervals (CrIs) were selected to reflect the assessments.

We conducted two types of meta-analyses. First, we conducted traditional pairwise meta-analyses using a random-effects model, through which the heterogeneities and publication biases among the trials were well anticipated before the Bayesian network meta-analysis. The analysis above evaluated the heterogeneities by the I^2^ statistic and judged the publication biases using funnel plots, and all the processes were performed in RevMan version 5.3.

A Bayesian network meta-analysis was conducted by using Aggregate Data Drug Information System (ADDIS, version 1.16.8). This software is based on the Bayesian framework and the Markov chain Monte Carlo method, which can evaluate *a priori* and process research data. The I^2^ statistic will be used to assess levels of the heterogeneity. Fixed-effects models will be used if the I^2^ value is 0.05, indicating good consistency. Iteration number will be set to 50,000; and the first 10,000 iterations for annealing will be set up to eliminate influences of the initial value. For indirect comparison, continuous outcomes will be calculated as standardized mean differences (SMDs), and binary outcomes will be calculated as ORs. Both types of effect sizes will be presented with 95% CrIs, and values of *p* < 0.05 will be regarded as statistically significant. The analysis of the network plot will show the evidence supporting the relationship between the included studies. Also, the result figures and network meta-analysis graphs will be provided.

## Results

### Study Identification and Selection

**Figure 1:**
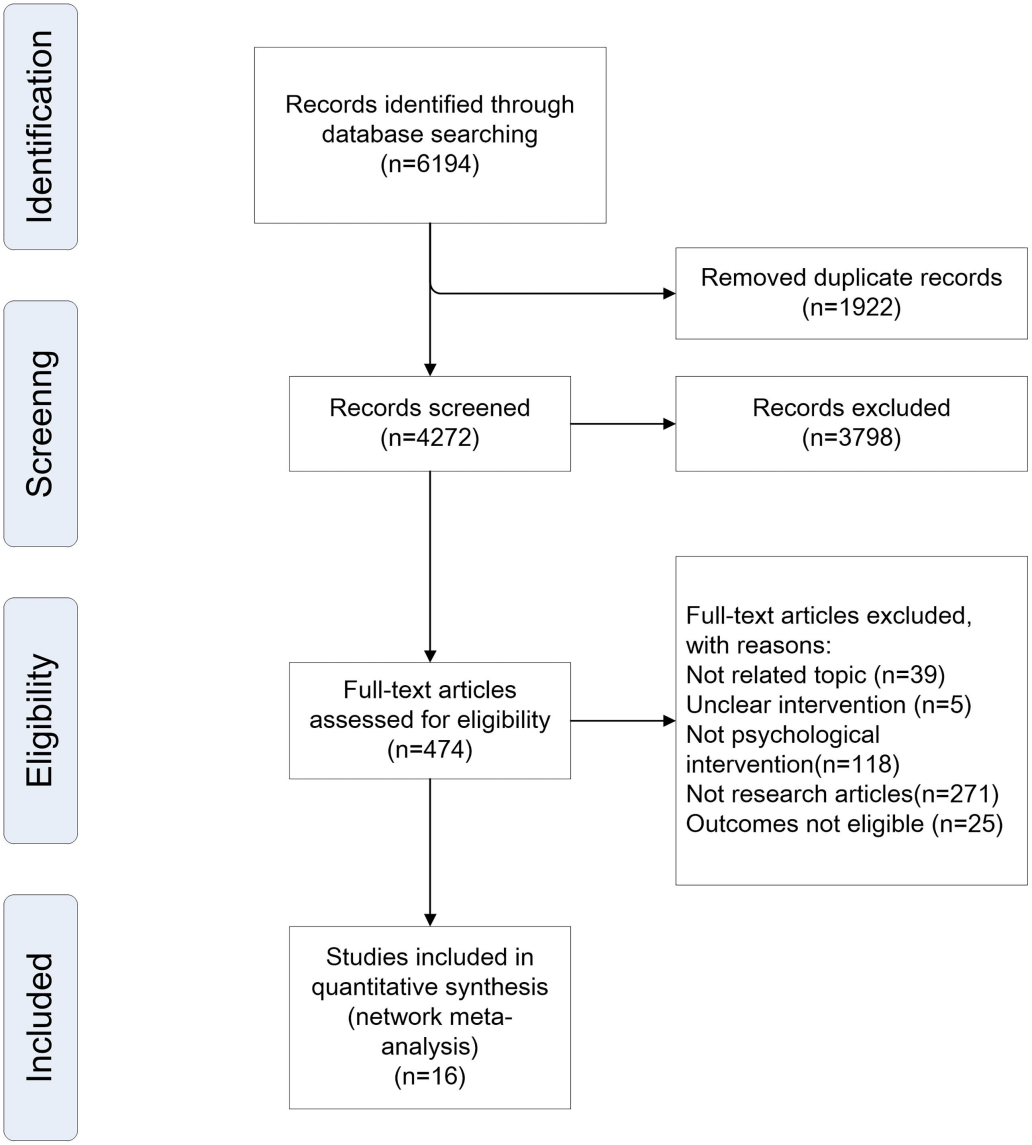
Study selection process.

In total, 6,194 citations published between 2019 and April 30, 2020, were identified by the search. Figure 1 shows the process of study selection. Eventually, 16 unique researches involving 1,147 unique patients were eligible for further analyses. The baseline characteristics of the studies were also extracted (Table 1).

### Quality Assessment of Included Studies

Due to the particularity of COVID-19, it is difficult to conduct RCT research as far. After final screening, all the experiments included in this systematic review are SCCS (nonrandomized) without any RCTs. So we utilized the Newcastle–Ottawa scale (NOS) with a slightly adapted version to match the needs of this study to evaluate the quality of SCCS studies (http://www.ohri.ca/programs/clinical_epidemiology/oxford.asp). The quality of the studies was evaluated by examining three items: patient selection, comparability of groups, and assessment of outcome. Studies were graded on an ordinal star scoring scale, with higher scores representing studies of higher quality. A study can be awarded a maximum of one star for each numbered item within the selection and exposure categories, and a maximum of two stars can be given for the comparability. The quality of each study was graded as either low quality (0–5) or high quality (6–9). The bias introduced in the studies included in this research was mainly attributed to the lack of community controls. The results of the risk of bias assessments for the SCCS studies are presented in Supplementary Figure 2.

### Meta-Analyses

There were a total of four network meta-analyses performed to compare and rank the included psychological interventions in four different psychological scales. The network of eligible comparisons for effectiveness consisted of 16 studies and 5 treatments. The consistency model was selected for the subsequent network analyses. Meanwhile, the inconsistency model was used to test consistency.

**Figure 2:**
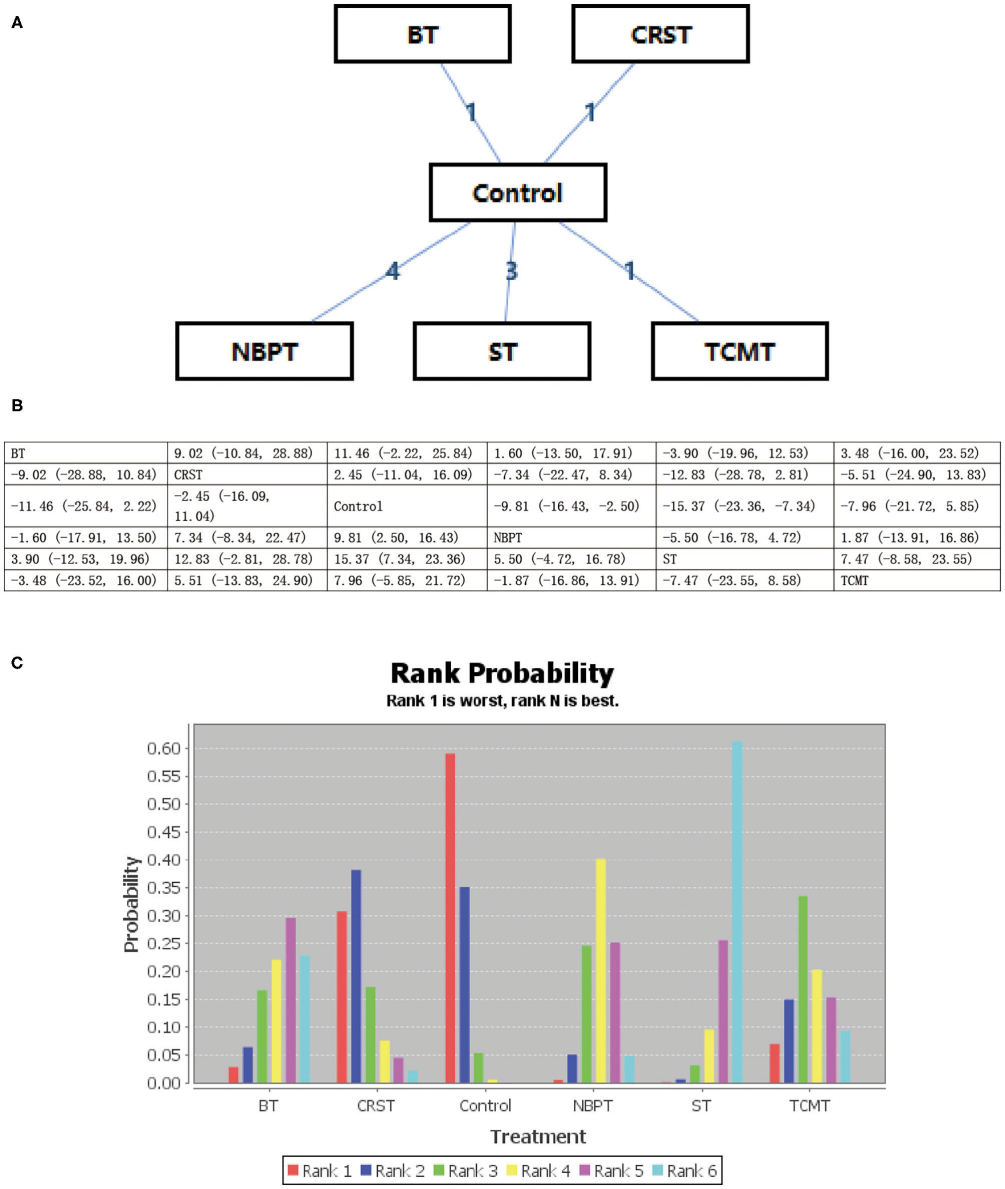
**(A–C)** Rank probability of effectiveness of psychological interventions assessed by SAS. ST, supportive therapy; BT, behavioral therapy; NBPT, nursing-based psychological therapy; TCMT, traditional Chinese medicine therapy; Control, before intervention; SAS, self-rating anxiety scale.

**Figure 3:**
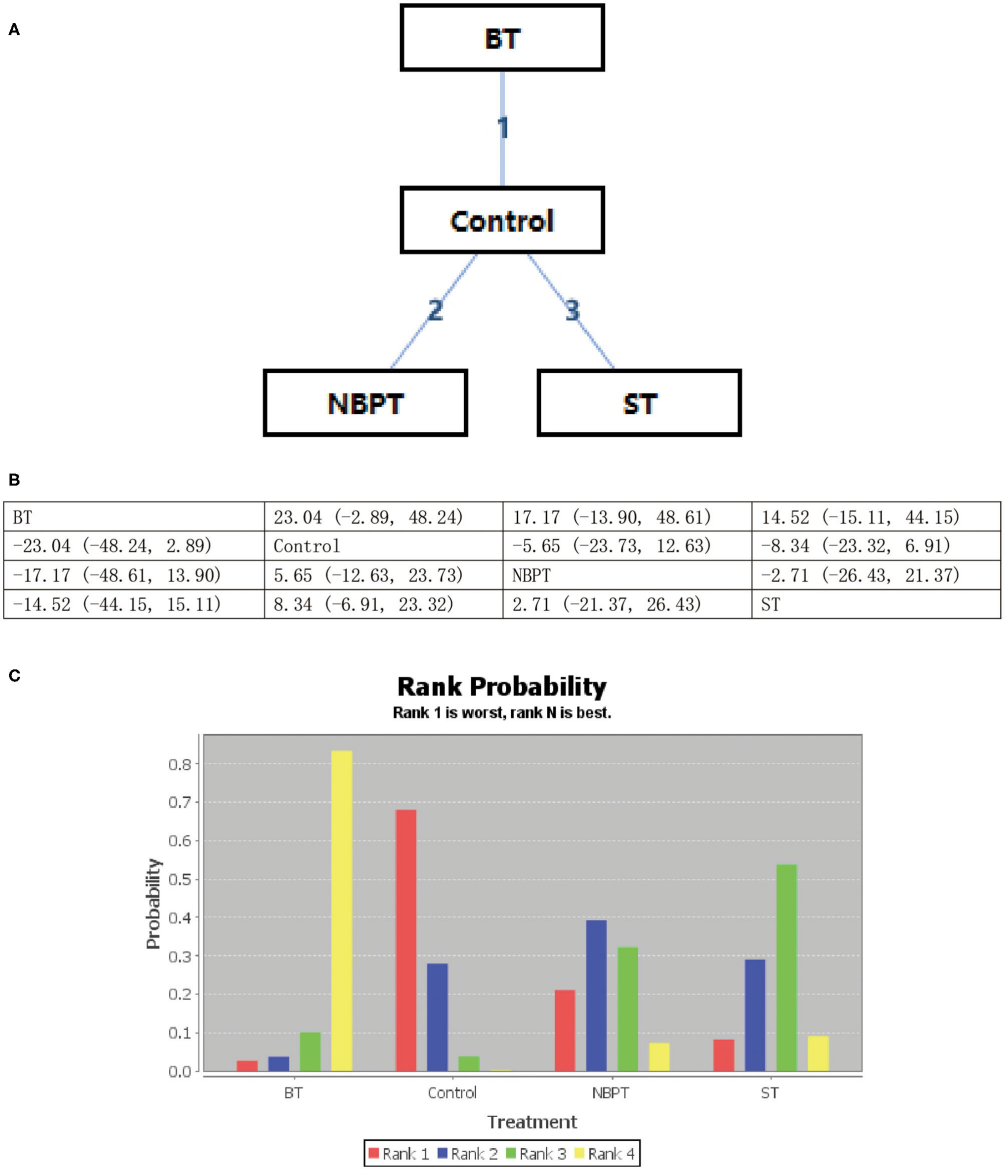
**(A–C)** Rank probability of effectiveness of psychological interventions assessed by SDS. ST, supportive therapy; BT, behavioral therapy; NBPT, nursing-based psychological therapy; Control, before intervention; SDS, self-rating depression scale.

**Figure 4:**
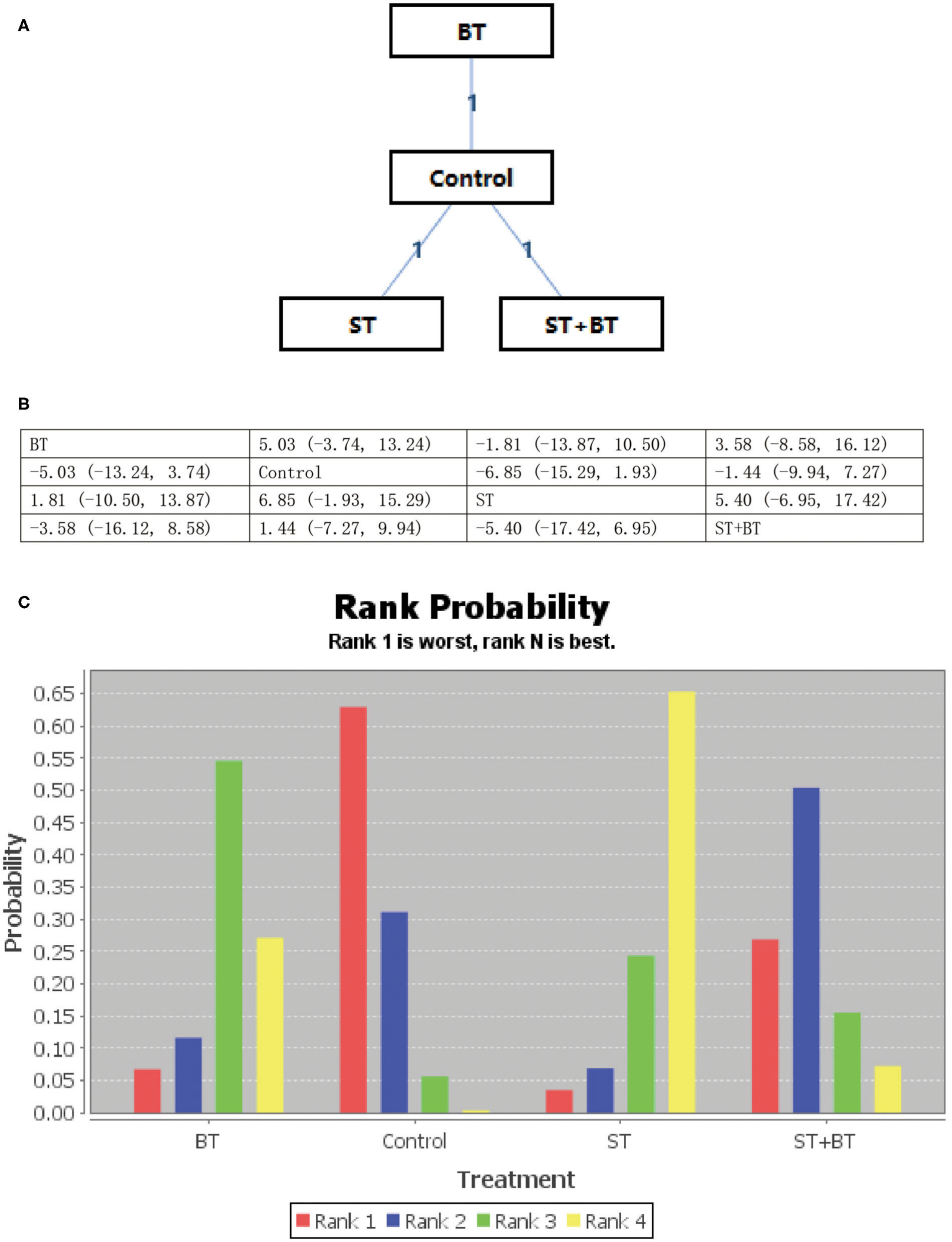
**(A–C)** Rank probability of effectiveness of psychological interventions assessed by PHQ-9. ST, supportive therapy; BT, behavioral therapy; ST+BT, combination of supportive therapy and behavioral therapy; Control, before intervention; PHQ-9, patient health questionnaire.

**Figure 5:**
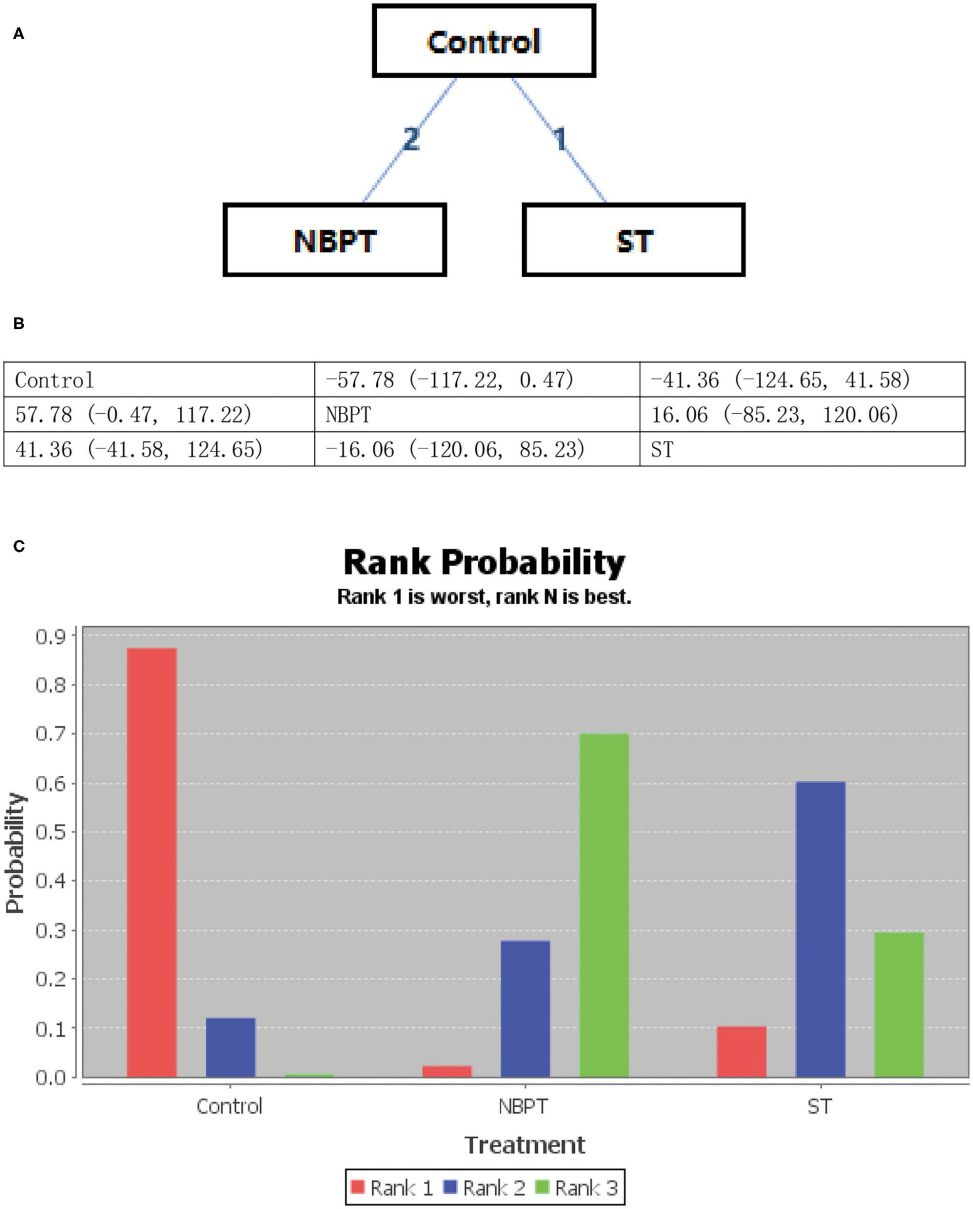
**(A–C)** Rank probability of effectiveness of psychological interventions assessed by SCL-90. ST, supportive therapy; NBPT, nursing-based psychological therapy; Control, before intervention; SCL-90, symptom checklist.

All psychological interventions were more beneficial than the control condition, but the best interventions on different scales are not completely consistent; also the rankings are also inconsistent. The results of our study indicated that ST was significantly more effective than the other treatments for reducing anxiety symptoms in SAS. Then the ranking is BT, NBPT, TCMT, and CRST. The ranking probability of treatments is presented in Figure 2. The second network meta-analysis was run to assess the most effective psychological interventions in SDS. We can see that BT was the best, followed by ST and NBPT. The specific network is presented in Figure 3. In terms of effectiveness in PHQ-9, group ST (SMD, 1.81; 95%CI 10.50, 13.87) were more effective than group BT. But interestingly, group ST+BT was the least effective one. The specific network is presented in Figure 4. In the final network meta-analysis, which was conducted to assess the effectiveness in SCL-90, only two kinds of psychological interventions (NBPT and ST) were included. They were both significantly more effective than before the intervention. And NBPT showed significantly more benefit than ST condition (SMD, 16.60; 95%CI, −85.23, 120.06) (Figure 5). Supplementary Figure 3 shows the results of pair-wise meta-analyses of compliance for each intervention.

## Discussion

This network meta-analysis included all available studies from 2019 to April 30, 2020, to analyze the effectiveness of psychological interventions for psychological crisis in people affected by COVID-19. After careful screening, a total of 16 articles were included in the study. Because of the rapid development of the epidemic, there are few published RCTs. Although all the studies were case studies and data of randomized controlled studies were lacking, most of our results had relatively high quality in terms of the NOS, and there was no obvious publication bias.

We can see that, in this study, the ranking of various interventions in different psychological scales was inconsistent, which indicated that different interventions may have different therapeutic effects on psychological problems. The ranking probability was primarily tied to direct and indirect effects that might provide robust evidence to support the results. Therefore, most of our conclusions were based on the ranking probability. But what can be found is that these interventions can effectively reduce the psychological crisis compared with before the intervention. First, there were five kinds of psychotherapies (ST, BT, NBPT, TCMT, and CRST) included in this network analysis that was assessed by SAS. Among them, ST showed a better effectiveness in the management of anxiety symptoms. ST is a commonly used and well-developed psychological intervention with a long history. The experiments we included in the study that used ST as a treatment were adjusted according to the particularity of the epidemic. They also showed significance in improving mental health assessed by PHQ-9 when compared with BT, or even the combination of ST and BT. However, these results may have been related to sample size. Then, based on our results, we found out that the most effective intervention for psychological crisis especially the depression symptoms according to the SDS test was BT, which is another commonly used non-pharmacological application defined as behavior-change intervention, including exercise or changes in daily activity to help deal with the psychological problems. And ST and NBPT were the second and third in reducing depression feelings as assessed by SDS. The last network meta-analysis only included two kinds of psychological interventions (NBPT and ST). And we found that NBPT was better than ST when measured by SCL-90. We did not standardize different measurement scales when conducting this network analysis, because each evaluation tool has special characteristics and focuses on different clinical manifestations of psychological problems. Therefore, it may be valuable to distinguish the clinical manifestations of psychological problems and adopt the best effective treatment options accordingly.

Due to the lack of understanding of COVID-19 in the first place, it is difficult to form a complete routine work process in a short period of time. Inadequate medical resources, insufficient medical protection and treatment measures, and the high infectivity of the virus have led to a sharp increase in the number of patients and a high mortality rate. As a result, the frontline medical staff and COVID-19 patients are suffering from psychological crisis to varying degrees (Huang and Zhao, 2020). It is easy to feel helpless and insecure and even to experience psychological problems such as anxiety, insomnia, fear, panic, blind disinfection, disappointment, irritability, aggressive behavior, and blind optimism (Duan and Zhu, 2020). Therefore, timely and effective psychological interventions can play a positive role in protecting the patients' physical and mental health (O'Donoghue et al., 2020). However, which intervention can better treat the psychological crisis has not been studied. Therefore, the results of this article are very meaningful.

## Conclusions

This research first evaluated the effectiveness of multiple psychological interventions for psychological crisis in people affected by COVID-19 *via* a Bayesian network meta-analysis and suggested potential benefit of psychological interventions for mental disorders caused by COVID-19 among all the affected people. Comprehensive analysis of the results indicated that ST was the most commonly used therapy and showed a better performance in all the measurement scales, with SAS and PHQ-9 in the first place and SCL-90 and SDS in the second. According to different assessment outcomes, ST, BT, and NBPT might be recommended for the COVID-19-affected people as their first-line treatment for managing psychological crisis. However, due to the limitations of case series studies, there is still a need for a larger sample size, especially high-quality RCTs and advanced analytic strategies in the future to confirm such conclusions.

### Strengths and Limitations

First, this is the first Bayesian network meta-analysis that comprehensively summarized all available evidences on the effectiveness of different psychological interventions in the treatment of psychological crisis during the COVID-19 pandemic. Second, it objectively recommended the best effective treatment options according to the clinical manifestations of psychological problems for people affected by COVID-19.

However, there were still some limitations included in this study: (1) all the included studies were case series. However, due to the rapid popularity of COVID-19, RCT or prospective studies have not been possible so far. (2) Although the language restriction was set as English and Chinese, we failed to include qualified English literature. However, as far as we know, there have been no reports of ethnic differences in the pathogenesis of COVID-19 so far. (3) We did not conduct subgroup analysis. (4) Due to the non-closed loop and few publications, the effectiveness of certain interventions may be exaggerated. (5) The ADDIS software is simple and convenient to operate, but it cannot be freely programmed, which may have some limits.

## Author Contributions

YY conceived the research and wrote the original draft. SS and SH were responsible for data selection and statistical analysis. CT participated in the search strategy development. YZ did a lot of work on the revision and finalization of the paper. HL participated in the design of data synthesis and to call the final determination when there still existed controversy after discussion. All authors listed have made a substantial, direct and intellectual contribution to the work, and approved it for publication.

## Conflict of Interest

The authors declare that the research was conducted in the absence of any commercial or financial relationships that could be construed as a potential conflict of interest.
