# Supplementary material for: Comparative Effectiveness of Multiple Psychological Interventions for Psychological Crisis in People Affected by Coronavirus Disease 2019: A Bayesian Network Meta-Analysis
Source: Front Psychol. 2021 Feb 22;12:577187. doi: 10.3389/fpsyg.2021.577187 (PMC7937808; doi:10.3389/fpsyg.2021.577187)
Supplement: Supplementary file 1 [file Data_Sheet_1.ZIP › Figure 1.docx]

**Table 1 Search terms on Pubmed**

| Term | Field |
| --- | --- |
| 2019 novel coronavirus disease | Mesh |
| COVID19 | Tittle/Abstract |
| COVID-19 pandemic | Tittle/Abstract |
| SARS-CoV-2 infection | Tittle/Abstract |
| COVID-19 virus disease | Tittle/Abstract |
| 2019 novel coronavirus infection | Tittle/Abstract |
| 2019-nCoV infection | Tittle/Abstract |
| Coronavirus disease 2019 | Tittle/Abstract |
| Coronavirus disease-19 | Tittle/Abstract |
| 2019-nCoV disease | Tittle/Abstract |
| COVID-19 virus infection | Tittle/Abstract |
| nCoV* | Tittle/Abstract |
| SARS2 | Tittle/Abstract |
| coronavirus | Mesh |
| 2019-nCoV | Tittle/Abstract |
| SARS-CoV-2 | Tittle/Abstract |
| Novel Coronavirus | Tittle/Abstract |
| 2019 novel coronavirus | Tittle/Abstract |
| Novel coronavirus 2019 | Tittle/Abstract |
| coronavirus 2019 | Tittle/Abstract |
| Corona Virus Disease-2019 | Tittle/Abstract |
| coronavirus disease 2019 virus | Tittle/Abstract |
| severe acute respiratory syndrome coronavirus 2 | Tittle/Abstract |
| Wuhan seafood market pneumonia virus | Tittle/Abstract |
| Wuhan coronavirus | Tittle/Abstract |
| Wuhan pneumonia | Tittle/Abstract |
| severe acute respiratory syndrome | Tittle/Abstract |
| COVID-2019 pneumonia | Tittle/Abstract |
| Infected Pneumonia | Tittle/Abstract |
| stress | Mesh |
| anxiety | Mesh |
| depression | Mesh |
| mental health | Mesh |
| psychiatry | Mesh |
| psychological intervention | Tittle/Abstract |
| psychological crisis | Mesh |
| Psycho* | Tittle/Abstract |
| Psychological Stress | Tittle/Abstract |
| Psychological Stresses | Tittle/Abstract |
| Stresses Psychological | Tittle/Abstract |
| Life Stress | Tittle/Abstract |
| Stress, Life | Tittle/Abstract |
| Stresses, Life | Tittle/Abstract |
| Stress, Psychologic | Tittle/Abstract |
| Psychologic Stress | Tittle/Abstract |
| Stressor Psychological | Tittle/Abstract |
| Psychological Stressor | Tittle/Abstract |
| Stressors Psychological | Tittle/Abstract |
| Mental Suffering | Tittle/Abstract |
| Suffering | Tittle/Abstract |
