# Supplementary material for: Comparative Effectiveness of Multiple Psychological Interventions for Psychological Crisis in People Affected by Coronavirus Disease 2019: A Bayesian Network Meta-Analysis
Source: Front Psychol. 2021 Feb 22;12:577187. doi: 10.3389/fpsyg.2021.577187 (PMC7937808; doi:10.3389/fpsyg.2021.577187)
Supplement: Supplementary file 1 [file Data_Sheet_1.ZIP › Figure 2.docx]

**Table 2 Summary of quality assessment of the included studies.**

|  | Selection | Comparability | Outcome |
| --- | --- | --- | --- |
| Chun-Yan Kuang 2020 |  |  |  |
| Cui Tian 2020 |  |  |  |
| Hong Chen 2020 |  |  |  |
| Li-Min Xing 2020 |  |  |  |
| Man-Ping Zeng 2020 |  |  |  |
| Wei Mi 2020 |  |  |  |
| Xia Li 2020 |  |  |  |
| Xia Xu 2020 |  |  |  |
| Xiao-Ping Huang 2020 |  |  |  |
| Xuan Zhou 2020 |  |  |  |
| Xue-Ying Li 2020 |  |  |  |
| Yang Zhang 2020 |  |  |  |
| Yan-Li Yang 2020 |  |  |  |
| Yan-Qiao Bao 2020 |  |  |  |
| Yan-Wen Dong 2020 |  |  |  |
| Ying Ren 2020 |  |  |  |
