# Supplementary figures and images for: Comparative Effectiveness of Multiple Psychological Interventions for Psychological Crisis in People Affected by Coronavirus Disease 2019: A Bayesian Network Meta-Analysis
Source: Front Psychol. 2021 Feb 22;12:577187. doi: 10.3389/fpsyg.2021.577187 (PMC7937808; doi:10.3389/fpsyg.2021.577187)

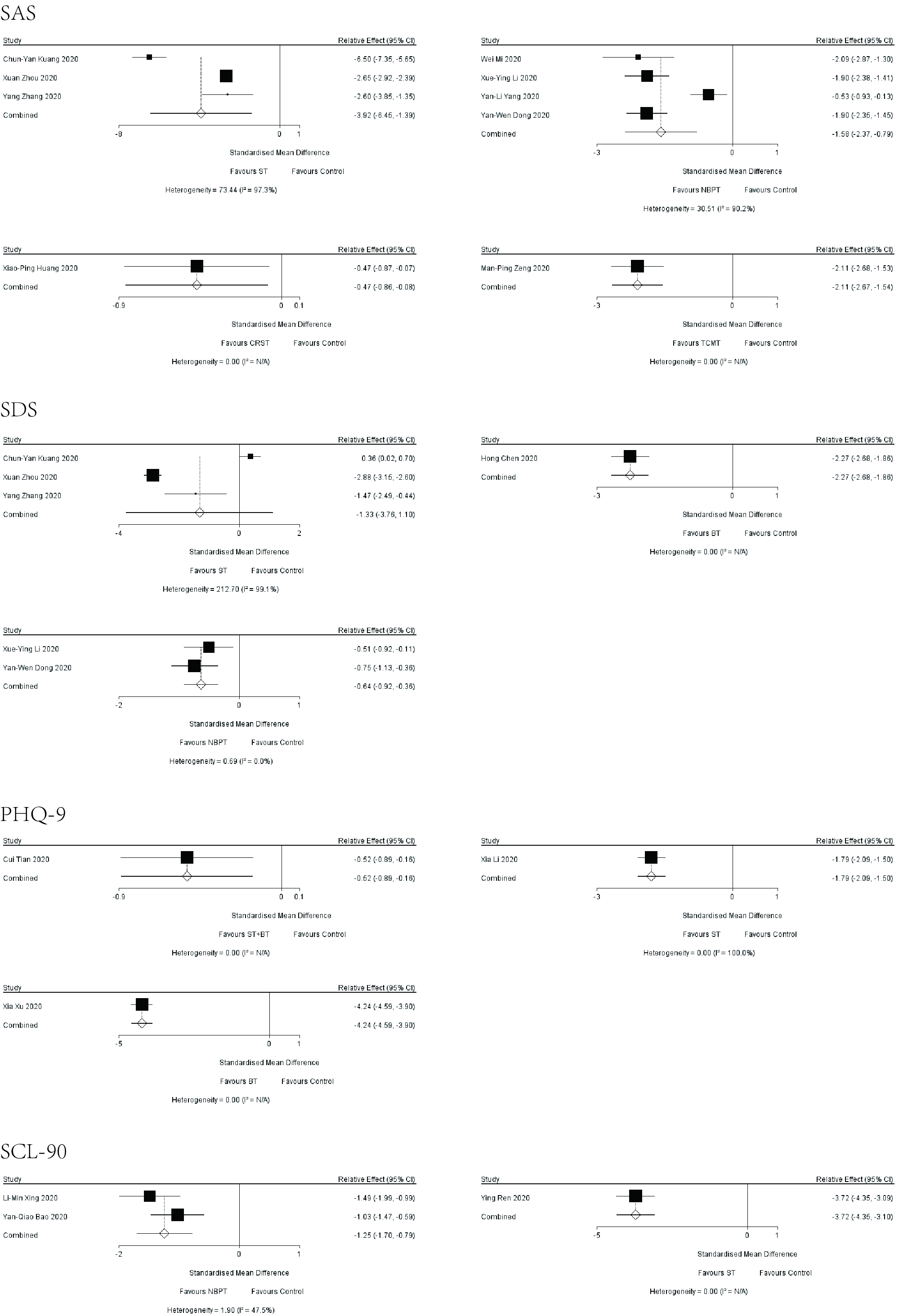

Supplement: Supplementary file 1 [file Data_Sheet_1.ZIP › Figure 3.tif]
